# Tuberculose cutanée à Bamako, Mali

**DOI:** 10.11604/pamj.2017.27.102.11577

**Published:** 2017-06-08

**Authors:** Adama Dicko, Ousmane Faye, Youssouf Fofana, Moussa Soumoutera, Siritio Berthé, Saidou Touré, Bekaye Traoré, Binta Guindo, Koureissi Tall, Alimata Keita, Lassine Kéita, Karim Coulibaly, Somita Keita

**Affiliations:** 1 Service de Dermatologie, Centre National d’Appui à la Lutte contre la Maladie, Bamako, Mali; 2 Faculté de Médecine de d’Odontostomatologie, Bamako, Mali; 3 Hôpital Régional de Sikasso, Bamako, Mali

**Keywords:** Tuberculose cutanée, scrofuloderme, Bamako, Mali, Cutaneous tuberculosis, scrofuloderma, Bamako, Mali

## Abstract

**Introduction:**

La tuberculose est la mycobactériose la plus fréquente en Afrique subsaharienne. La localisation cutanée est rare et sous diagnostiquée à cause de son polymorphisme clinique et la faiblesse du plateau technique. Le but de cette étude était de décrire les aspects épidémiologiques, cliniques, histopathologiques de la tuberculose cutanée à Bamako (Mali).

**Méthodes:**

De janvier 1991 à décembre 2008 nous avons réalisé une étude transversale descriptive. L’étude s’est déroulée dans le service de Dermatologie du Centre National d’Appui à la lutte contre la Maladie et le service de Pneumo-phtisiologie au l’hôpital du Point G. Ont été inclus dans l’étude les cas de tuberculose confirmés par l’histologie et ou la biologie.

**Résultats:**

Sur 4269 dossiers, 61 cas de tuberculose cutanée étaient recensées (1,43%). Les hommes représentaient 59% des cas (36 malades) et les femmes 41 % soit (25 cas); soit un sex-ratio de 1,44. L’âge des malades variait de 3 mois à 61 ans pour une moyenne de 27,56 ± 36 ans. La durée d’évolution était en moyenne de 10,9 ± 10 mois. Les formes cliniques recensées étaient le scrofuloderme (41 cas), la forme ulcéreuse (13 cas), la forme verruqueuse (4 cas), et le lupus tuberculeux (3 cas). La tuberculose était associée au VIH dans 7 cas, à la lèpre dans 3 cas.

**Conclusion:**

La tuberculose cutanée est sous diagnostiquée au Mali. Des efforts sont nécessaires pour améliorer l’accessibilité et le plateau technique des services spécialisés, pour mener une étude approfondie interdisciplinaire sur cette pathologie.

## Abstract (en)

**Introduction:**

Tuberculosis is the most common mycobacteriosis in sub-Saharan Africa. Cutaneous tuberculosis is rare and underdiagnosed due to its clinical polymorphism and to the smallness of technical equipment. This study aims to describe the epidemiological, clinical, histopathological aspects of cutaneous tuberculosis in Bamako (Mali).

**Methods:**

We conducted a cross-sectional descriptive study from January 1991 to December 2008. The study was performed in the Department of Dermatology at the National Center for Disease Control and in the Department of Pneumophtisiology at the Hospital of Point G. The patients with tuberculosis confirmed by histological and/or biological examination were included in the study.

**Results:**

Out of 4269 patients? medical records, 61 cases of cutaneous tuberculosis were identified (1.43%). Men accounted for 59% of the cases (36 patients) and women 41% (25 cases); sex-ratio was 1.44. The age of the patients ranged from 3 months to 61 years, with an average age of 27.56 ± 36 years. The average length of follow-up was 10.9 ± 10 months. The identified clinical forms were scrofuloderma (41 cases), ulcerative form (13 cases), verrucous form (4 cases), and tuberculous Lupus (3 cases). Tuberculosis was associated with HIV in 7 cases, with leprosy in 3 cases.

**Conclusion:**

Cutaneous tuberculosis is underdiagnosed in Mali. Efforts are needed to improve the accessibility and the technical equipment available in the Departments, in order to conduct an extensive interdisciplinary study on this pathology.

## Introduction

La tuberculose est une maladie infectieuse chronique et contagieuse due au complexe Mycobacterium. C’est un problème de santé publique dans de nombreux pays en développement, selon l’organisation mondiale de la santé (OMS) 9,6 millions de personnes auraient contracté la tuberculose en 2014 [1]. Elle est l’une des principales causes de décès (1,5 million) dans le monde selon l’OMS [2]. En Afrique, avec l’avènement du VIH-sida certaines localisations extra pulmonaires ont vu leur nombre croitre [3]. La peau représente 2,1% des cas, cette localisation est caractéristique par son polymorphisme, le retard au diagnostic et l’assiduité au traitement. La découverte d’une forme cutanée peut guider au dépistage d’un foyer pulmonaire latent. Le but de cette étude était de décrire les aspects épidémiologiques, cliniques, histopathologiques de la tuberculose cutanée à Bamako (Mali).

## Méthodes

De janvier 1991 à décembre 2008 nous avons mené une étude transversale descriptive sur des dossiers de malades vus pour tuberculose cutanée. L’étude s’est déroulée dans les plus grand centre de prise en charge de tuberculose de la capitale. Il s’agit des services de Dermatologie du Centre National d’Appui à la lutte contre la Maladie (CNAM) et le service de Pneumo-phtisiologie au CHU du Point G. A partir des dossiers cliniques, de compte rendu d’histophatologie nous avons recensé tous les cas. Le diagnostic de tuberculose cutanée était basé sur les données cliniques, l’histologie et la biologie. Ont été non inclus tous les dossiers incomplets. Les données épidémiologiques (sexe, âge, profession, ethnie, lieu de résidence), cliniques (motifs de consultation, caractère clinique des lésions, la durée d’évolution, le siège des lésions), biologiques (NFS, une radio pulmonaire, statut VIH, IDR), les données histologie ont été recueillies à partir des dossiers cliniques. Toutes ces données ont été recueillies à l’aide d’un questionnaire, puis saisies et analysées avec le logiciel SPSS 12.0.

## Résultats

**Figure 1:**
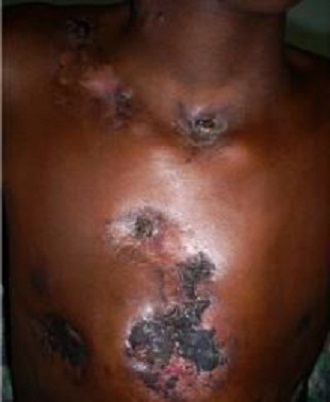
Scrofuloderme localisation cervicale et poitrine

**Figure 2:**
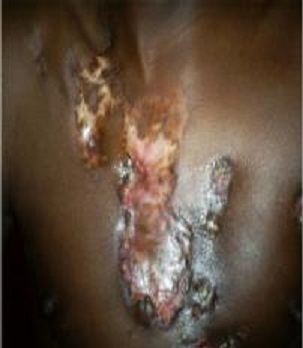
Scrofuloderme localisation poitrine et cervicale

**Figure 3:**
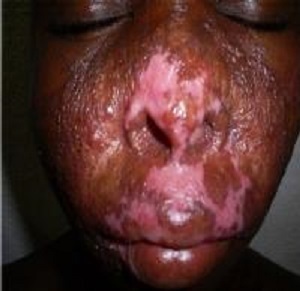
Lupus tuberculeux facial

Durant la période d’étude, sur 4269 dossiers, nous avons recensés 61 cas de tuberculose cutanée soit 1,43%. Les hommes représentaient 59% des cas (36 malades) et les femmes 41% soit (25 cas); soit un sex-ratio de 1,44. L’âge moyen était de 27 ans avec des extrêmes de 3 mois et 61 ans. Les enfants (3 mois-15 ans) représentaient 18% des cas (11/61). Les professions les plus représentées étaient les ménagères avec 13,11% suivi des commerçants 9,84% et les élèves étudiants 6,56%. Parmi eux 65,6% proviennent des zones rurales de Koulikoro (22 cas), Kayes (12 cas) et Mopti (12 cas). Les principaux motifs de consultation étaient l’adénopathie dans 60,6% cas, une fistulisation dans (19,6%) des cas, La durée d’évolution de la maladie variait de 1,5 mois à 109,6 mois avec une durée moyenne de 10,9 ± 10 mois. Les signes d’imprégnations bacillaires étaient l’anorexie retrouvée 35 fois, amaigrissement 46 fois et l’asthénie 38 fois. Au plan clinique la localisation des lésions était: cervicale et sus claviculaire dans 42,6% (26/61), axillaire 9,8% (6/61), bras et dos 8,2% (5/61) chacun (Tableau 1). Les formes clinques retrouvées étaient le scrofuloderme dans 67,2% soit 41 cas (Figure 1, Figure 2), la tuberculose ulcéreuse dans 21,3% (13 cas), la forme verruqueuse dans 6,6% (4 cas), et le lupus tuberculeux dans 4,9% soit 3 cas (Figure 3). La tuberculose était associée au VIH dans 7 cas (11,48%), à la lèpre dans 3 cas (4,92%).

**Tableau 1:** Répartition selon le résultat de la biopsie

Forme clinique	Fréquence	Pourcentage
Scrofuloderme/adénite	41	67,2
Lupus tuberculeux	3	4,9
Tuberculose verruqueuse	4	6,6
Tuberculose ulcéreuse	13	21,3
Total	61	100

## Discussion

Le but de ce travail était de décrire les aspects épidémiologiques, cliniques et biologiques de la tuberculose cutanée dans deux grandes structures spécialisées dans sa prise en charge: il s’agit du service de pneumologie du point G et du service de dermatologie du CNAM. Nos exigences méthodologiques dans le diagnostic excluaient systématiquement tous les cas probables (« faux positifs ») qui auraient pu être considérés à tort comme tuberculose cutanée. Néanmoins cette étude nous donne un aperçu sur la tuberculose cutanée à Bamako au Mali malgré son caractère de recueil des données rétrospectives. Elle représente 1,4% dans notre étude pendant cette période. Les malades atteints étaient 36 hommes pour 25 femmes, avec un sex-ratio de 1,44. L’atteinte cutanée représente environ 2% des formes extra-pulmonaires qui sont estimées à 18% selon les rapports fournis par le Programme National de Lute contre la Tuberculose (PNLT) au Mali [4]. A Dakar, elle représentait 4,7% des motifs de consultation [5]. Le sexe masculin était le plus atteint. Les tendances actuelles observées ne montraient généralement aucune prédominance d’un sexe par rapport à l’autre. L’atteinte des sujets jeunes dans notre étude est une donnée classique de la tuberculose en Afrique [5, 6]. Toutes les couches socio-professionnelles étaient représentées. Les plus touchées étaient ceux qui proviennent des zones rurales (65,6%) cela s’explique par le bas niveau socioéconomique. Les facteurs de risque classiquement identifiés à l’heure actuelle sont: la promiscuité, la pauvreté, le manque d’hygiène, l’infection à VIH et le mauvais état nutritionnel du sujet.

La maladie avait évoluée en moyenne 10 mois, jusqu’à 109 mois pour certains. Cela expliquait en partie la sévérité du tableau clinique observé chez certains patients. Cette longue évolution s’expliquerait par plusieurs facteurs: La prédominance des adénites tuberculeuses et le caractère indolent de cette forme qui ne motive pas une consultation immédiate: l’ignorance et l’analphabétisme; l’éloignement et le manque de moyens financiers limitaient l’accessibilité des centres spécialisés. Certaines association on rendue sévère aussi le tableau clinique de nos patients il s’agit de 7 cas associe au VIH 1 et 3 cas associés à une lèpre multi bacillaire. Le VIH est connu comme facteur aggravant le tableau clinique et responsable d’une diminution progressive de l’immunité, source d’infections opportunistes parmi lesquelles, la tuberculose est la plus fréquente [2]. La lèpre affaiblissait aussi l’organisme et donc le rend plus vulnérable au Bacille de Koch [7]. Parmi les formes cliniques le scrofuloderme a été la forme la plus notée dans notre étude soit (47,50%) contrairement aux autres formes également retrouvées comme le lupus tuberculeux. Cela concorde avec les résultats des travaux antérieurs effectués à Dakar et à Madagascar [5, 6]. La localisation cervicale (37,70%) observée dans notre série avait déjà été rapportée à Dakar et au Mali [5, 8]. Si cette localisation élective cervicale est classique, d’autres comme les fistules anales, sont rares. Il s’agit en général, de suppuration traînante. L’examen histologique de la pièce de fistulectomie nous a permis de poser le diagnostic. Dans notre série, nous n’avons noté que 2 cas d’atteinte génitale. Il s’agit d’une forme particulière rare responsable de stérilité observée généralement au stade de complications.

## Conclusion

La tuberculose cutanée est sous diagnostiquée au Mali. Des efforts sont nécessaires pour améliorer l’accessibilité et le plateau technique des services spécialisés, pour mener une étude approfondie interdisciplinaire sur cette pathologie.

### Etat des connaissances actuelle sur le sujet

- Problème de santé publique dans de nombreux pays dans le monde;
- L’une des principales causes de décès dans le monde;
- La peau représente 2,1% des localisations.

### Contribution de notre étude à la connaissance

- Fréquence hospitalière de la tuberculose cutanée au Mali;
- Son association avec la lèpre dans 3 cas.

## Conflits d’intérêts

Les auteurs ne déclarent aucun conflit d’intérêt.
